# Supplementary material for: Improving wellness: Defeating Impostor syndrome in medical education using an interactive reflective workshop
Source: PLoS One. 2022 Aug 4;17(8):e0272496. doi: 10.1371/journal.pone.0272496 (PMC9352101; doi:10.1371/journal.pone.0272496)

**S1 Table:**

Title: Session Time-Table

Caption: Outline of reflective workshop schedule and PowerPoint Presentation

Impostor syndrome is a psychological term characterized by chronic feelings of self-doubt and internalized fear of being exposed as an intellectual fraud. Sufferers are unable to internalize and own their successes, accomplishments, competence, or skills. Overall, they believe themselves to be less intelligent and competent than others perceive them to be. Implications of imposter syndrome in academic medicine includes correlations with burnout, decreased performance, health ailments and psychological defects.

**Description of Session:**

1. **Pre-survey:** Participants will complete Young Imposter Syndrome & Competence Quiz. This will provide awareness and baseline for the participants.
2. **Animated Video Presentation:** The participants will watch a short U-tube video in which Elizabeth Cox describes the psychology behind the imposter syndrome, and what you can do to combat it. [TED-Ed Animation by Sharon Coleman
3. **Imposter Syndrome presentation**: A presentation on the concept, literature and background on Imposter syndrome will be given
4. **Small Group discussion on combating Imposter in the learning environment:** Imposter syndrome scenarios will be used. Participants in small groups will deliberate on corrective intervention strategies on Thought Questions below
5. **Whole group discussion “You can still have an impostor moment, but not an impostor life”:** Each group will give report to the whole group with further discussions
6. **Post-survey:** A short perception, knowledge and behavior-based survey using Kirkpatrick level 1,2,3 will be provided at the end of the session

**Session Time-Table**

| **Activity** | **Activity** | Time |
| --- | --- | --- |
| Pre-survey: | Participants complete a short survey awareness and knowledge regarding Imposter syndrome. This will provide baseline for the participants: worksheet | 10 minutes |
| Animated Video Presentation | TED-Ed Animation by Sharon Coleman | 5 minutes |
| Imposter Syndrome presentation: | PowerPoint presentation on Characteristics, How did this happen, Features; Why it sucks, High profile public examples, Competence types, Strategies to address | 15 minutes |
| Group Activity 1 | Participants will deliberate on thought questions to suggest possible strategies & recommendations: google docs | 10 minutes |
| Group Activity 2 | Participants will deliberate on scenarios suggest possible interventions to help; google docs | 10 minutes |
| Post-survey | For wrap up, the participants will complete the short survey to objectively determine any changes in perception from the video-based intervention: worksheet | 10 minutes |

<https://www.ted.com/talks/elizabeth_cox_what_is_imposter_syndrome_and_how_can_you_combat_it?language=en>


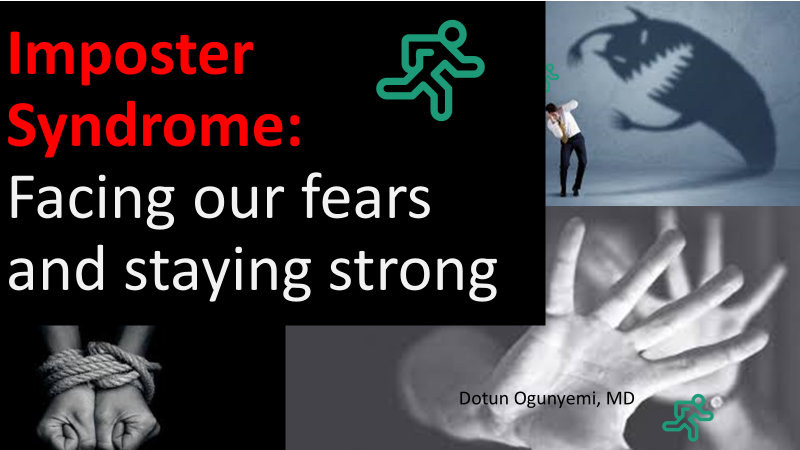


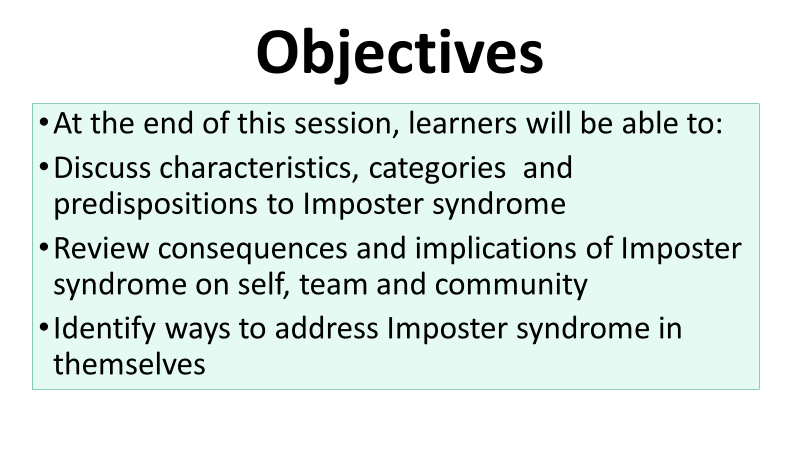


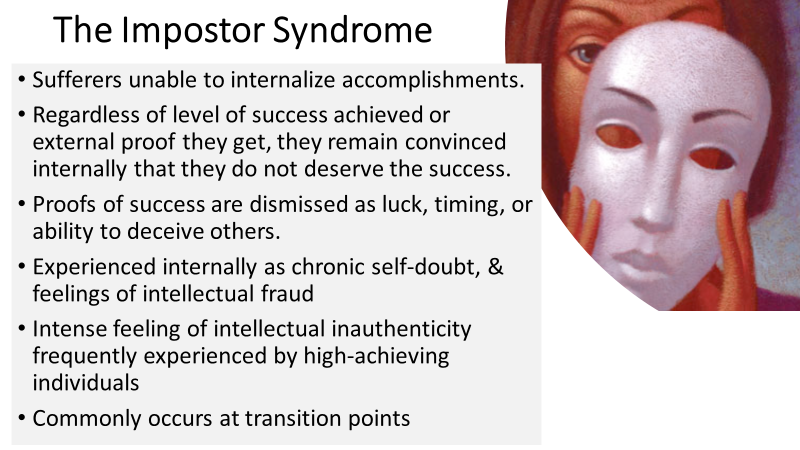


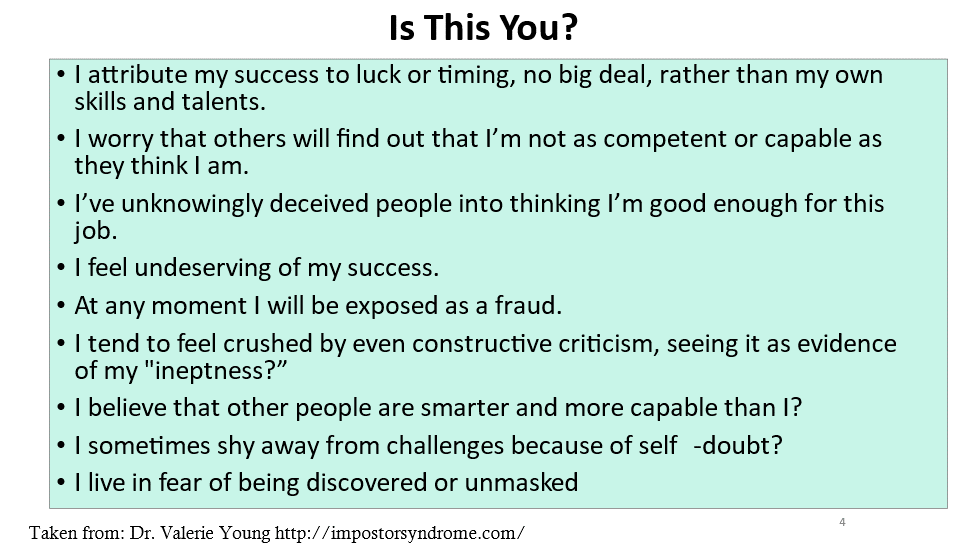


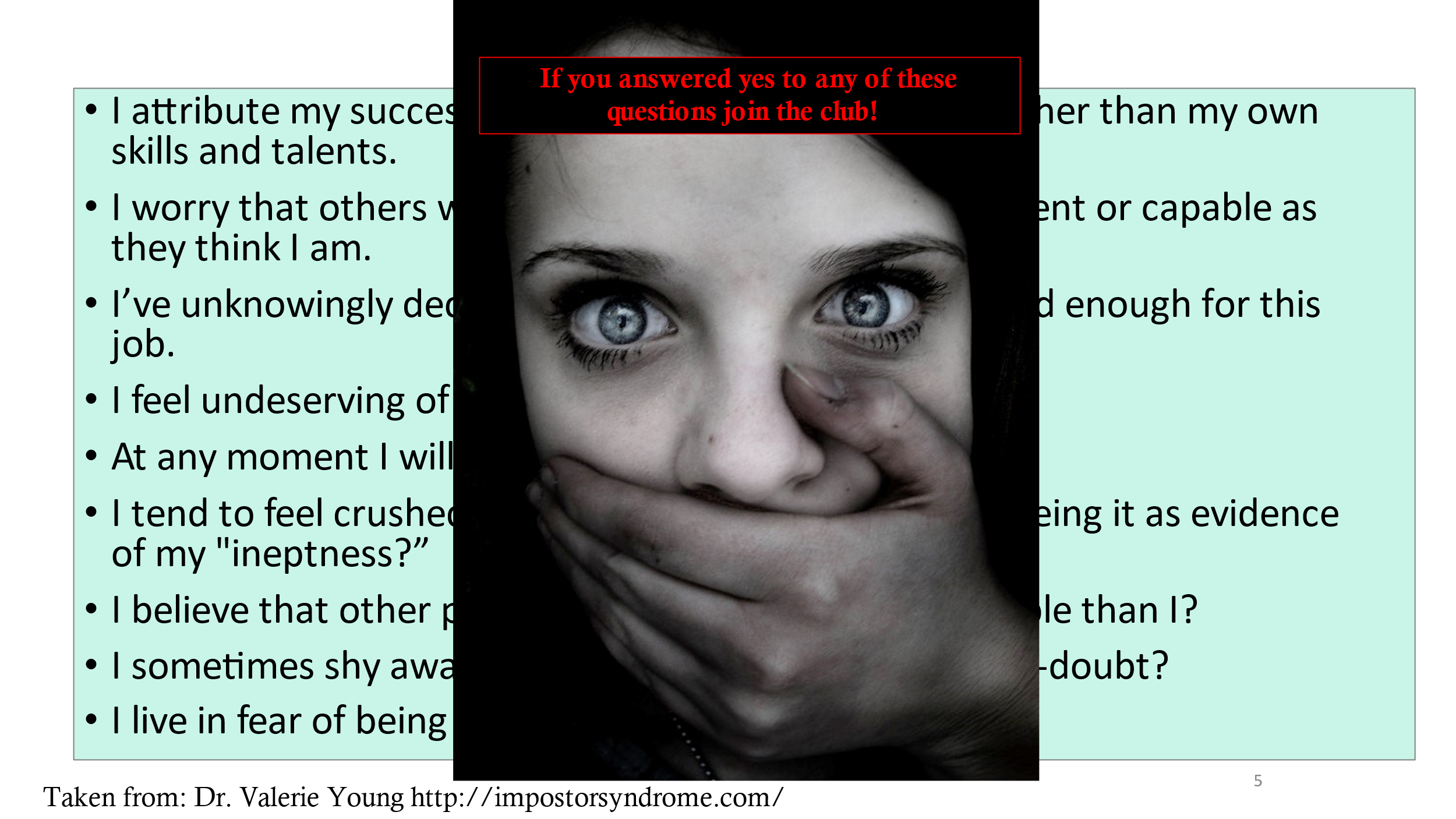


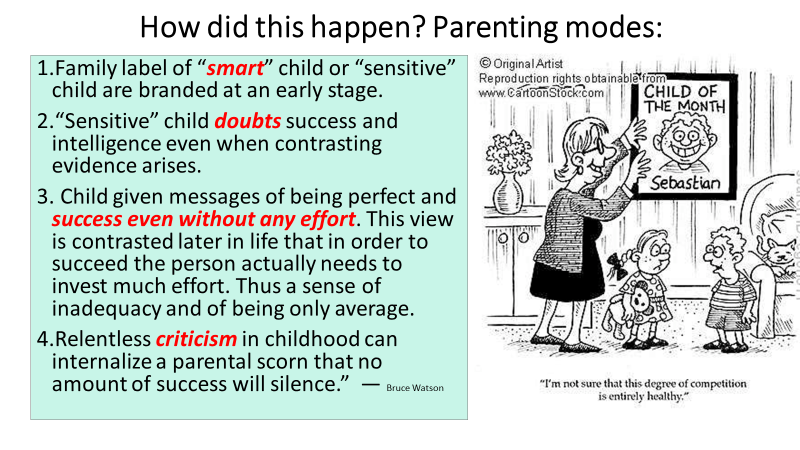


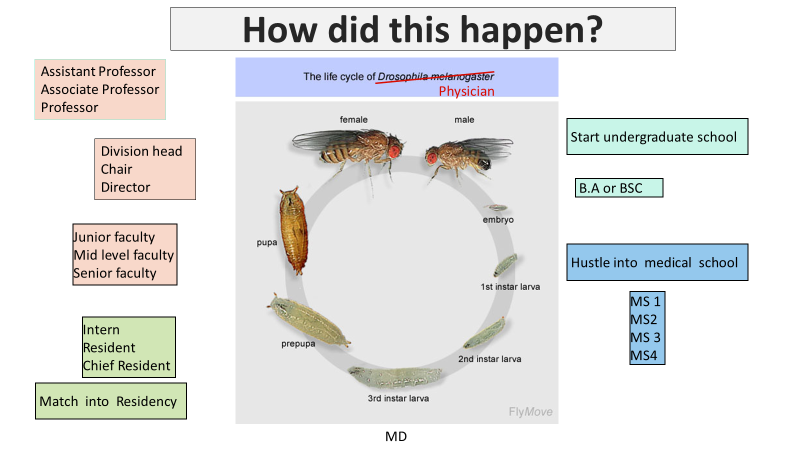


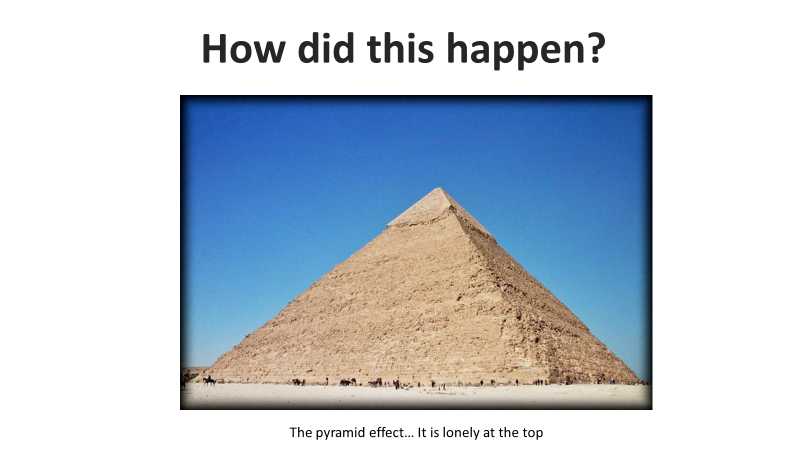


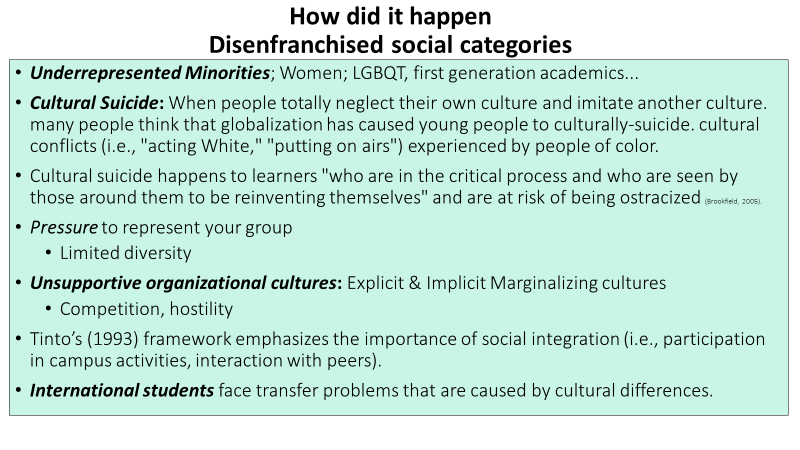


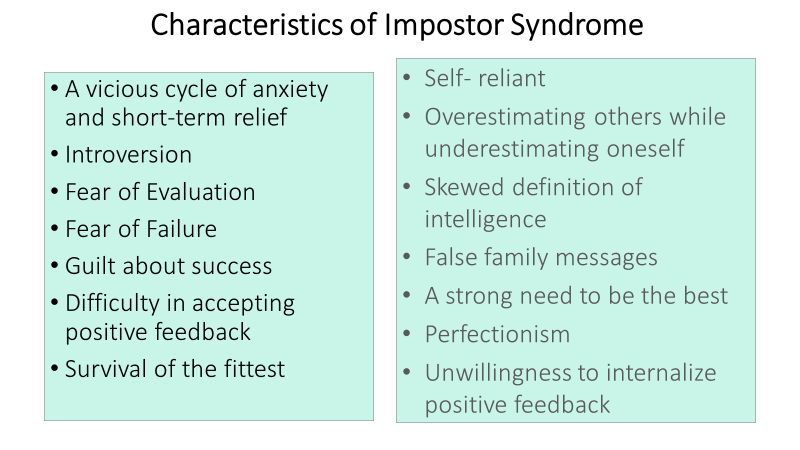


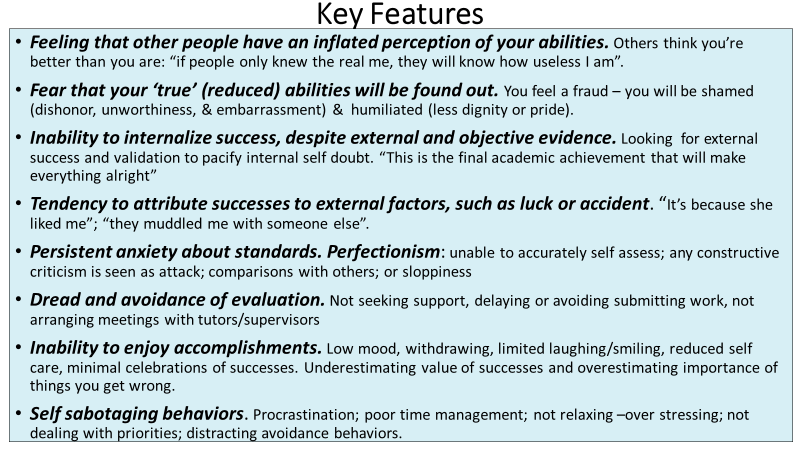


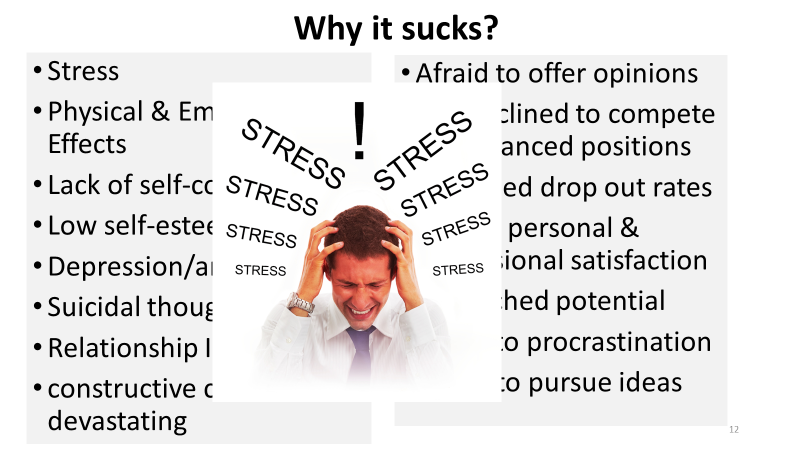


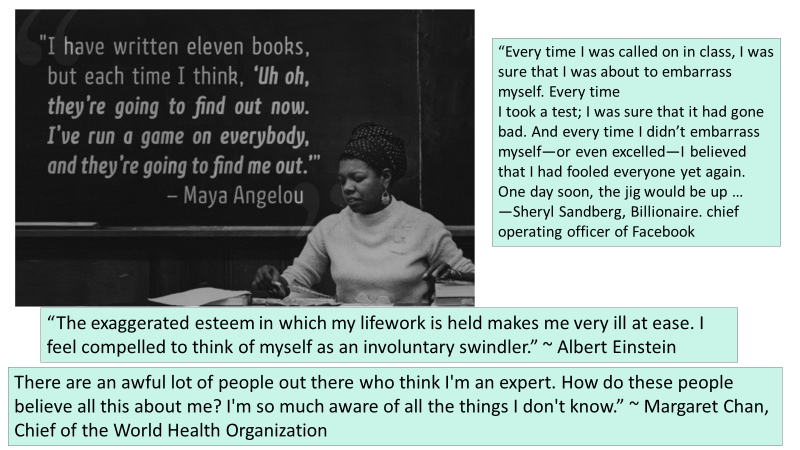


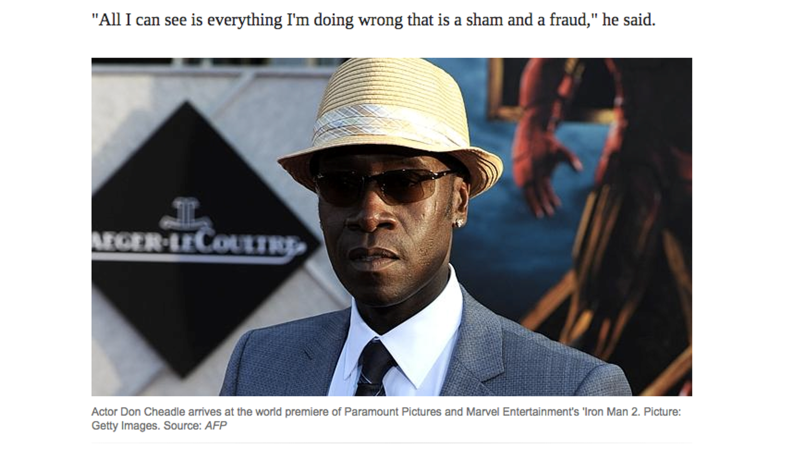


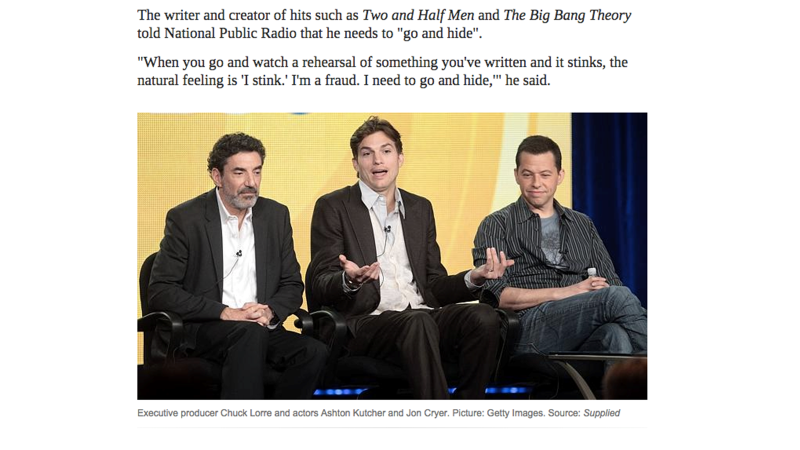


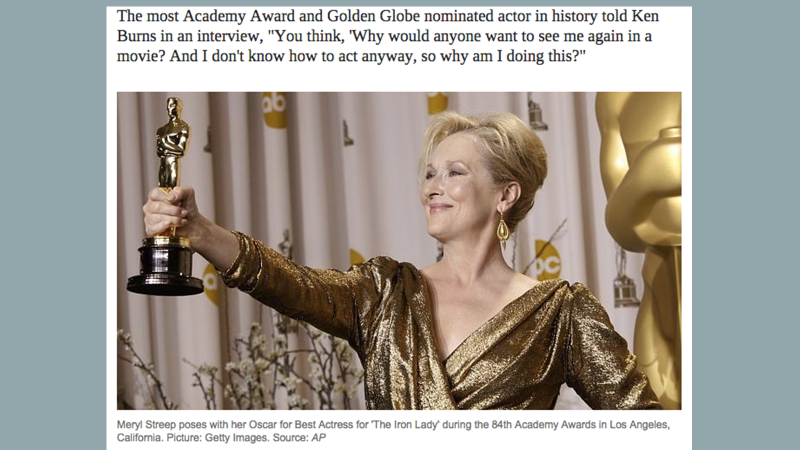


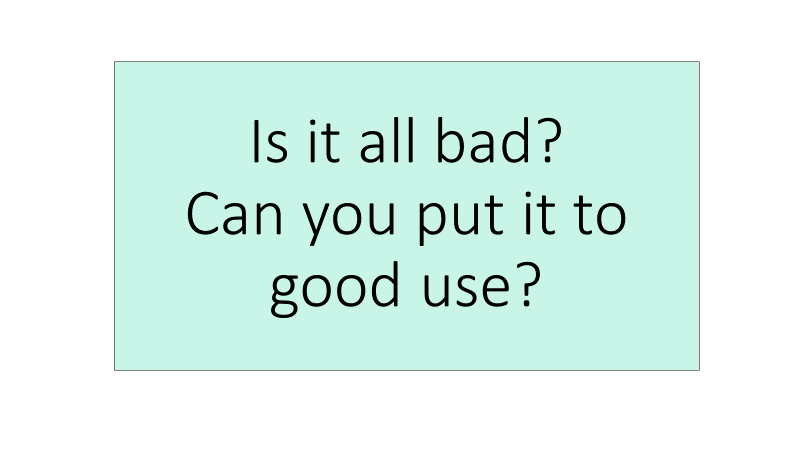


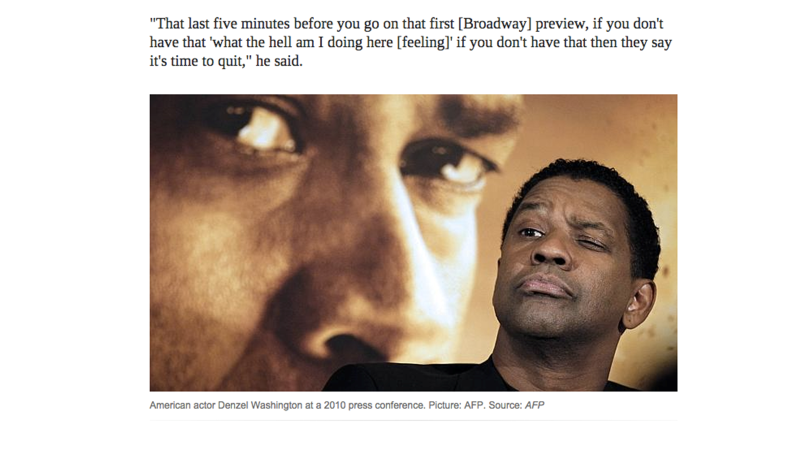


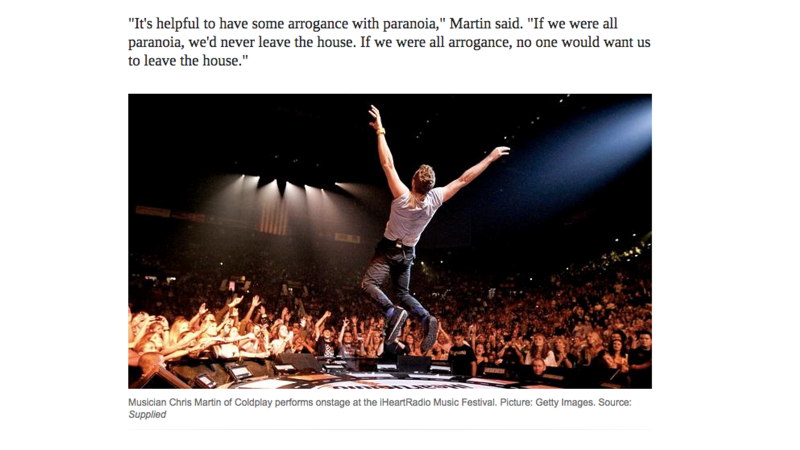


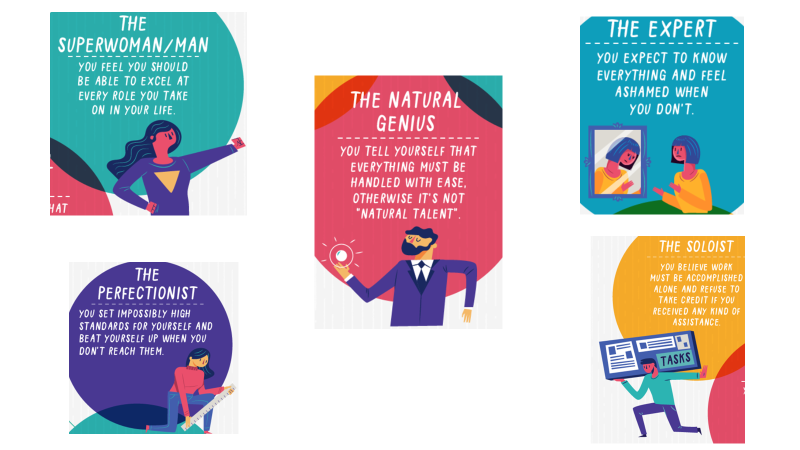


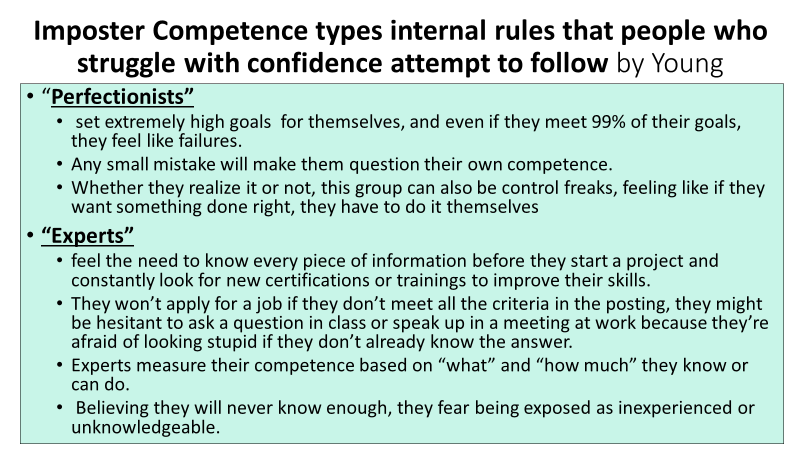


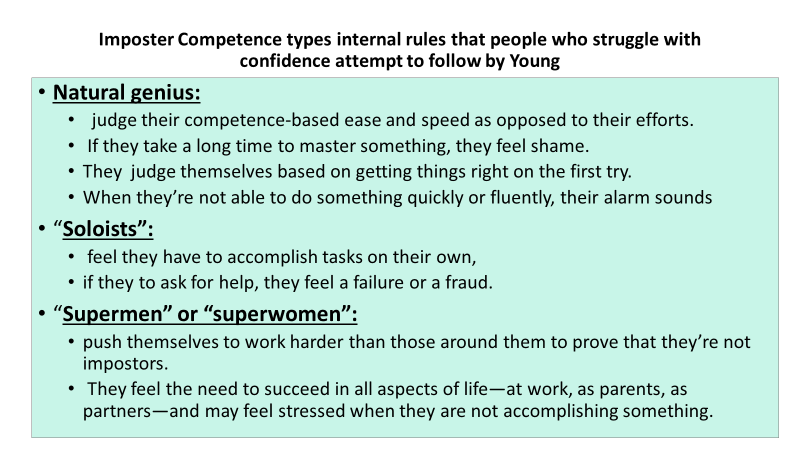


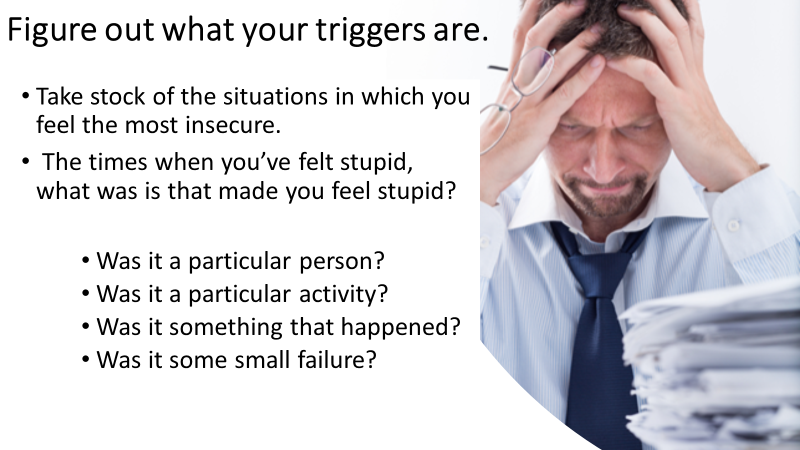


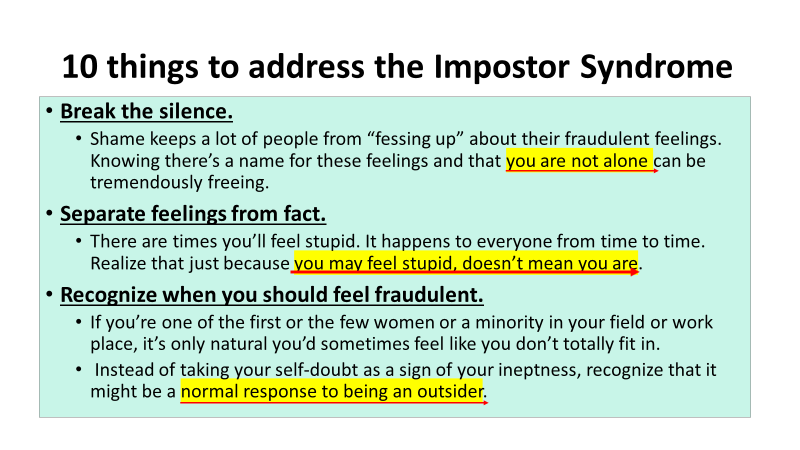


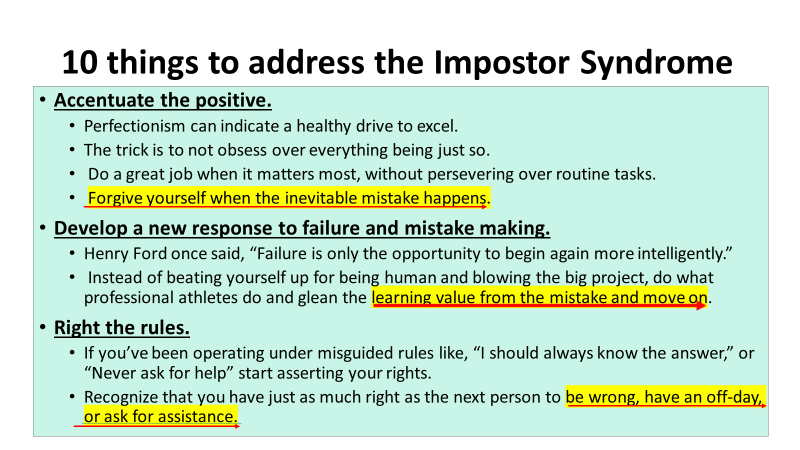


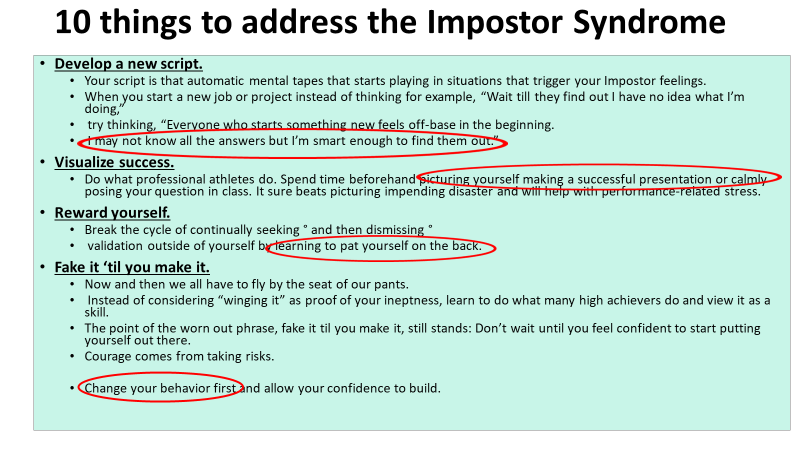


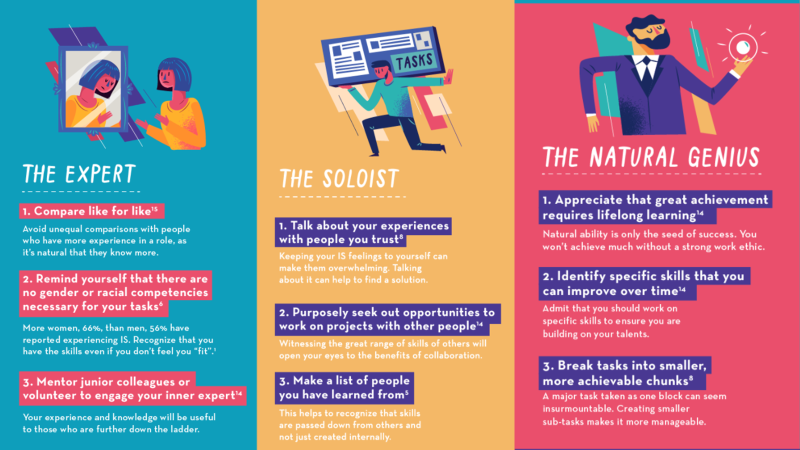


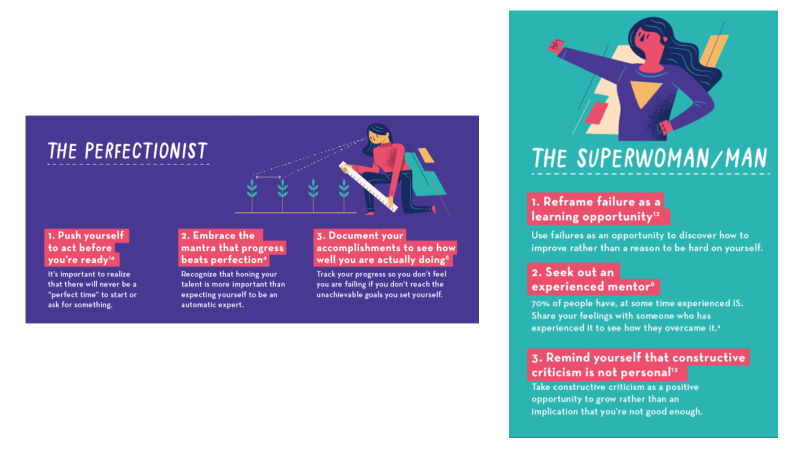


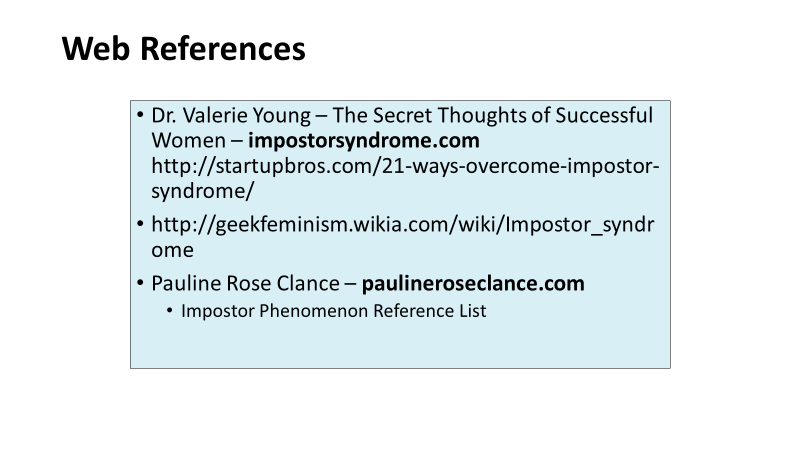

Supplement: S1 Table — Outline of reflective workshop schedule and PowerPoint Presentation. (DOCX) [file pone.0272496.s002.docx]
