# Supplementary material for: Improving wellness: Defeating Impostor syndrome in medical education using an interactive reflective workshop
Source: PLoS One. 2022 Aug 4;17(8):e0272496. doi: 10.1371/journal.pone.0272496 (PMC9352101; doi:10.1371/journal.pone.0272496)
Supplement: S2 Table — Description of the 5 competence subtypes within Impostor syndrome. (DOCX) [file pone.0272496.s003.docx]

**S2 Table:**

Title: Impostor Syndrome Competence Subtypes

Caption: Description of the 5 competence subtypes within Impostor syndrome

| **Competence subtype** | **Characteristics** | **Mitigating Strategies** |
| --- | --- | --- |
| **Perfectionist:**  set impossible standards and beat yourself, when it is not achieved | - set extremely high goals for themselves, and even if they meet 99% of their goals, they feel like failures. - Any small mistake will make them question their own competence. - Whether they realize it or not, this group can also be control freaks, feeling like if they want something done right, they have to do it themselves | - Push yourself to act before you are ready; there is never a perfect time to act - Embrace the fact that progress better than perfection - Document your accomplishments to show how you are doing - Track your progress so that you don’t feel you are failing; be aware how much you have achieved even if you do not reach the unachievable goal that you set |
| **Experts:**  expect to know everything, and ashamed if you don’t | - feel the need to know every piece of information before they start a project and constantly look for new certifications or trainings to improve their skills. - They won’t apply for a job if they don’t meet all the criteria in the posting, - They might be hesitant to ask a question in class or speak up in a meeting at work because they’re afraid of looking stupid if they don’t already know the answer. - Experts measure their competence based on “what” and “how much” they know or can do. - Believing they will never know enough; they fear being exposed as inexperienced or unknowledgeable. | - Avoid unequal comparison with people who have more experience than you in a role or position - Remember that based on your training and experience, you have the skills and competencies for your position; race or gender or ‘ism” is not a competency. - Mentors others to engage your inner expert. Your experience and knowledge will be valuable for those farther down the ladder |
| **Natural genius:** everything must be handled with ease | - judge their competence-based ease and speed as opposed to their efforts. - If they take a long time to master something, they feel shame. - When they’re not able to do something quickly or fluently, their alarm sounds - They judge themselves based on getting things right on the first try. | - Appreciate that great achievement requires life-long learning - Identify specific skills that you can improve over time - Break tasks into smaller, more achievable chunks |
| **Soloist:**  work must be accomplished alone | - feel they have to accomplish tasks on their own, - If they to ask for help, they feel a failure or a fraud | - Talk about your experience with people that you trust - Purposely seek out opportunities to work on projects with other people - Remind yourself and be cognizant of people that you have learned from or those that have helped you reach your goal |
| **Super-person:**  You should be able to excel at every role in your life | - push themselves to work harder than those around them to prove that they’re not impostors. - They feel the need to succeed in all aspects of life—at work, as parents, as partners—and may feel stressed when they are not accomplishing something. | - Reframe failure as a learning opportunity - Seek out an experienced mentor - Remind yourself that constructive criticism |
