# Supplementary material for: Improving wellness: Defeating Impostor syndrome in medical education using an interactive reflective workshop
Source: PLoS One. 2022 Aug 4;17(8):e0272496. doi: 10.1371/journal.pone.0272496 (PMC9352101; doi:10.1371/journal.pone.0272496)
Supplement: S1 Appendix — Survey materials completed by participants prior to reflective workshop. (DOCX) [file pone.0272496.s004.docx]

**S1 Appendix:**

Title: Pre-Test Survey

Caption: Survey materials completed by participants prior to reflective workshop

**Pre-Test**

**Circle the title that applies to you:**

Staff Basic Science Faculty Clinical Faculty Resident Medical Student Other

Specific title:

**Optional:** Gender: F M (circle 1) Race:

**Knowledge Questions:**

**Which of the following is TRUE of Imposter syndrome?**

1. Experienced externally as chronic self-doubt, & feelings of intellectual fraud
2. Intense feeling of intellectual inauthenticity frequently experienced by low-achieving individuals
3. Experienced internally as chronic self-assurance & feelings of intellectual over-achievement.
4. Require enormous amount of external proof to be convinced internally that they deserve the success that they have achieved.
5. Proofs of success are dismissed as luck, timing, or ability to deceive others.

Answer:

1. If you exhibit any of the following, which would be the most likely sign that you may have Imposter syndrome?
2. I attribute my success to my own skills and talents.
3. I sometimes shy away from challenges because of my expertise.
4. I have already been exposed as a fraud.
5. I tend to accept constructive criticism, seeing it as evidence for growth.
6. I’ve unknowingly deceived people into thinking I’m good enough for this job.

Answer:

1. Which of the following public figures have openly acknowledged the symptoms of Imposter syndrome? Select the combination that most applicable.

Actress Meryl Streep: winner of Oscar for Best Actress

Actor Don Cheadle: Iron man, Avengers

Maya Angelou: Famed author and poet

Sheryl Sandberg: Billionaire, chief operating officer of Facebook

Albert Einstein: famed Inventor & Scientist

1. None of the public figures
2. All of the public figures
3. Don Cheadle & Meryl Streep
4. Sheryl Sandberg
5. Maya Angelou & Albert Einstein

Answer:

**Match the Imposter type with the appropriate behavior**

1. Super-person Answer:
2. Soloists Answer:
3. Natural genius Answer:
4. Expert Answer:
5. Perfectionist Answer:
6. If they take a long time to master something, they feel shame.
7. They won’t apply for a job if they don’t meet all the criteria in the posting.
8. Any small mistake will make them question their own competence.
9. if they to ask for help, they feel a failure or a fraud.
10. They feel the need to succeed in all aspects of life.

Match the behavior with one of the 10 things you can use to break the Imposter syndrome

1. Forgive yourself when the inevitable mistake happens Answer:
2. Okay to be wrong, have an off-day, or ask for assistance. Answer:
3. Change your behavior first and allow your confidence to build Answer:
4. You may feel stupid, doesn’t mean you are Answer:
5. Break the silence.
6. Separate feelings from fact
7. Recognize when you should feel fraudulent.
8. Fake it till you make it.
9. Accentuate the positive
10. Develop a new response to failure and mistake making.
11. Right the rules
12. Develop a new script.
13. Visualize success.
14. Reward yourself.

**Young Imposter Syndrome Quiz**

**This survey determines if you have Imposter Syndrome**

**Circle YES or NO**

1. Do you secretly worry that others will find out that you're not as bright and capable as they think you are?

YES NO

2. Do you sometimes shy away from challenges because of a nagging self-doubt?

YES NO

3. Do you tend to chalk your accomplishments up to being a "fluke," "no big deal" or the fact that people just "like" you?

YES NO

4. Do you hate making a mistake, being less than fully prepared, or not doing things perfectly?

YES NO

5. Do you tend to feel crushed even by constructive criticism, seeing it as evidence of your "ineptness?"

YES NO

6. When you do succeed, do you think "Phew, I fooled them this time, but I may not be so lucky next time?"

YES NO

7. Do you believe that other people (students, colleagues, competitors) are smarter and more capable than you?

YES NO

8. Do you live in fear of being found out, discovered, or unmasked?

YES NO

**This survey determines what “competence types”—or internal rules that people who struggle with Imposter Syndrome attempt to follow**

**In answering the following questions think about your response using a Likert scale of:**

**1=strongly disagree, 2= disagree; 3= neutral; 4= agree; 5= strongly agree**

**Type 1**

1. Have you ever been accused of being a micromanager?

1 2 3 4 5

1. Do you have great difficulty [delegating](https://www.themuse.com/advice/the-10-rules-of-successful-delegation)? Even when you’re able to do so, do you feel frustrated and disappointed in the results?

1 2 3 4 5

1. When you miss the (insanely high) mark on something, do you accuse yourself of “not being cut out” for your job and ruminate on it for days?

1 2 3 4 5

1. Do you feel like your work must be 100% perfect, 100% of the time?

1 2 3 4 5

**Type 2**

1. Do you stay later at the office than the rest of your team, even past the point that you’ve completed that day’s necessary work?

1 2 3 4 5

1. Do you get stressed when you’re not working and find downtime completely wasteful?

1 2 3 4 5

1. Have you left your hobbies and passions fall by the wayside, sacrificed to work?

1 2 3 4 5

1. Do you feel like you haven’t truly earned your title (despite numerous degrees and achievements), so you feel pressed to work harder and longer than those around you to prov Are you used to excelling without much effort?

1 2 3 4 5

**Type 3:**

1. Do you have a track record of getting “straight A’s” or “gold stars” in everything you do?

1 2 3 4 5

1. Were you told frequently as a child that you were the “smart one” in your family or peer group?

1 2 3 4 5

1. Do you dislike the idea of [having a mentor](https://www.themuse.com/advice/4-myths-finding-having-a-mentor), because you can handle things on your own?

1 2 3 4 5

1. When you’re faced with a setback, does your confidence tumble because not performing well provokes a feeling of shame?

1 2 3 4 5

1. Do you often avoid challenges because it’s so uncomfortable to try something you’re not great at?

1 2 3 4 5

**Type 4:**

1. Do you firmly feel that you need to accomplish things on your own?

1 2 3 4 5

1. “I don’t need anyone’s help.” Does that sound like you?

1 2 3 4 5

1. Do you frame requests in terms of the requirements of the project, rather than your needs as a person?

1 2 3 4 5

**Type 5:**

1. Do you shy away from applying to job postings unless you meet every single educational requirement?

1 2 3 4 5

1. Are you constantly seeking out trainings or certifications because you think you need to improve your skills in order to succeed?

1 2 3 4 5

1. Even if you’ve been in your role for some time, can you relate to feeling like you still don’t know “enough?”

1 2 3 4 5

1. Do you shudder when someone says you’re an expert?
   1 2 3 4 5

**STOP PRETEST HERE**
