# Supplementary material for: Improving wellness: Defeating Impostor syndrome in medical education using an interactive reflective workshop
Source: PLoS One. 2022 Aug 4;17(8):e0272496. doi: 10.1371/journal.pone.0272496 (PMC9352101; doi:10.1371/journal.pone.0272496)
Supplement: S2 Appendix — Materials for Group activities utilized during reflective workshop. (DOCX) [file pone.0272496.s005.docx]

Group Activity

Group Activity 1

In breakout groups discuss one of the thought questions below & deliberate on 3 strategies or recommendations:

**Thought Questions:**

***Please checkmark your groups’ selection***

1. What are the 3 most effective or feasible actions that an institution can take to help decrease the impact of imposter syndrome on the students?
2. What are the 3 best changes a supervisor or teacher can take to decrease the effect of Imposter syndrome on the learning environment?
3. What are the 3 best actions that we can take to decrease the effect of our own imposter syndrome on our own health, emotions, and family?
4. What are 3 most compassionate and empathic behaviors that the healthcare team to decrease the impact of imposter syndrome on patient centered care, patient satisfaction, and patient safety?

**Please list your 3 strategies of your group:**

**Strategy 1:**

**Strategy 2:**

**Strategy 3:**

**Comments:**

Group Activity 2

In breakout groups discuss one of the scenarios below & deliberate on 3 strategies or recommendations:

**Scenario for reflections 1::**

***Please checkmark your groups’ selection***

A first-year medical student was born in Valenzuela and immigrated to United States with her mother when she was 5 years old, because her father had been killed by gangs. She is first in her family to go to college. She has 3 siblings, and she is the oldest. She had to work since age 14 years to help financially support her mum who worked 3 jobs. She is a non-traditional student who went to community college worked in fast food restaurant to raise enough money to attend medical school. She feels she is not good enough. She believes she will never know enough, and fear being exposed as untrainable, unknowledgeable and should not be in medical school.

How can we help her?

**Please list your 3 strategies of your group:**

**Strategy 1:**

**Strategy 2:**

**Strategy 3:**

**Comments:**

**Scenario for reflections 2:**

Dr Superior is the Program Director of Neurosurgery, he is also an editor of a prestigious journal and on the board of directors of the State Medical Board. However, residents complain that Dr Superior gets upset if they miss any little detail. He changes course many times, then everyone has to pivot with his new directive. The program coordinators feel limited because they have to pass every little detail by Dr Superior and cannot make decisions. Dr Superior works long hours, because he also has grants and other national very important academic projects he is working. Last week, he missed his daughter’s graduation from elementary school and the week before he could not make his wife’s birthday dinner.

How can we help Dr Superior?

**Please list your 3 strategies of your group:**

**Strategy 1:**

**Strategy 2:**

**Strategy 3:**

**Comments:**

**Scenario for reflections 3:**

Ms. Genius is a coordinator in department of Medical Education. She grew up believing she was good at everything. However, she was given a new project to do, and she was not able to accomplish as quickly or fluently as required. She now has doubts that she cannot accomplish tasks on her own, feel that if she asks for help, she is a failure or a fraud. Constructive criticism offered by her supervisor was devastating because it confirmed what she felt even though he did praise all the other things she did well, but those positive compliments don’t matter. She is refusing to give her opinions because she does not want to be wrong. She is getting frustrated, withdrawn, stressed and depressed.

How can we help Ms. Genius?

**Please list your 3 strategies of your group:**

**Strategy 1:**

**Strategy 2:**

**Strategy 3:**

**Comments:**
