# Supplementary material for: Improving wellness: Defeating Impostor syndrome in medical education using an interactive reflective workshop
Source: PLoS One. 2022 Aug 4;17(8):e0272496. doi: 10.1371/journal.pone.0272496 (PMC9352101; doi:10.1371/journal.pone.0272496)
Supplement: S3 Appendix — Survey materials completed by participants after reflective workshop. (DOCX) [file pone.0272496.s006.docx]

Title: Post-Test Survey

Caption: Survey materials completed by participants after reflective workshop

**Imposter Syndrome Post Perception Survey**

**Circle the title that applies to you:**

Staff Basic Science Faculty  Clinical Academic Faculty Clinical Faculty Resident Medical Student Other

Specific title/class/location:

Gender: F M (circle 1) Race:

**In answering the following questions think about respond using a Likert scale of:**

**1=strongly disagree, 2= disagree; 3= neutral; 4= agree; 5= strongly agree**

**Perception questions**

Respond using a Likert scale of: 1=strongly disagree, 2= disagree; 3= neutral; 4= agree; 5= strongly agree

1. I now realize that I have Imposter syndrome that has impeded me from rising to my full potential

1 2 3 4 5

1. It is clear to me that my imposter syndrome may be responsible for stress, low self-esteem, frustration or other negative emotions in my life

1 2 3 4 5

3) I can see how my imposter syndrome may have negatively affected my team at work or school

1. 2 3 4 5

4) I now recognize how the imposter syndrome of my supervisor or faculty has limited my ability to be most productive and engaged

1. 2 3 4 5

5) I now realize how my imposter syndrome may have affected my relationships and my family

1. 2 3 4 5

**Behavior changing questions**

6) I feel that I have learned some skills from this workshop that can help me deal with my imposter syndrome

1. 2 3 4 5

7) I feel that I can help make changes in my workplace or institution that are necessary to decrease the effect of imposter syndrome in our workplace or learning environment

1. 2 3 4 5

**Reflection questions**

8) Which of the following do you think contributed to your imposter syndrome? Circle all that may apply.

Parents’ expectations or rearing styles: Underrepresented Minorities: Women: LGBQT:

First generation academics: Academic Rat Race: International Transfers: Academic Transitions

Unsupportive organizational cultures: Foreign Accent: Changed academic or career plans:

Others:

**Knowledge Questions:**

**Which of the following is TRUE of Imposter syndrome?**

1. Experienced externally as chronic self-confidence, & feelings of intellectual fraud
2. Intense feeling of intellectual inauthenticity frequently experienced by low-achieving individuals
3. Experienced internally as chronic self-assurance & feelings of intellectual over-achievement
4. Require external proof to be convinced internally that they deserve the success that they have achieved
5. Proofs of success are dismissed as luck, timing, or ability to deceive others

Answer:

1. If you exhibit any of the following, which would be the most likely sign that you may have Imposter syndrome?
2. I attribute my success to my own skills and talents
3. I sometimes shy away from challenges because of my ignorance
4. I have already been exposed as a fraud
5. I tend to accept constructive criticism, seeing it as evidence for growth
6. I’ve unknowingly deceived people into thinking I’m good enough for this job.

Answer:

1. Which of the following public figures have openly acknowledged the symptoms of Imposter syndrome? Select the combination that most applicable.

Actress Meryl Streep: winner of Oscar for Best Actress

Actor Don Cheadle: Iron man, Avengers

Maya Angelou: Famed author and poet

Sheryl Sandberg: Billionaire, chief operating officer of Facebook

Albert Einstein: famed Inventor & Scientist

1. None of the public figures
2. All of the public figures
3. Don Cheadle & Meryl Streep
4. Sheryl Sandberg
5. Maya Angelou & Albert Einstein

Answer:

**Match the Imposter type with the appropriate behavior**

1. Super-person Answer:
2. Soloists Answer:
3. Natural genius Answer:
4. Expert Answer:
5. Perfectionist Answer:
6. If they take a long time to master something, they feel shame
7. They won’t apply for a job if they don’t meet all the criteria in the posting
8. Any small mistake will make them question their own competence
9. if they to ask for help, they feel a failure or a fraud
10. They feel the need to succeed in all aspects of life

Match the behavior with one of the 10 things you can use to break the Imposter syndrome

1. Forgive yourself when the inevitable mistake happens Answer:
2. Okay to be wrong, have an off-day, or ask for assistance. Answer:
3. Change your behavior first and allow your confidence to build Answer:
4. You may feel stupid, doesn’t mean you are Answer:
5. Break the silence
6. Separate feelings from fact
7. Recognize when you should feel fraudulent
8. Fake it till you make it
9. Accentuate the positive
10. Develop a new response to failure and mistake making
11. Right the rules
12. Develop a new script
13. Visualize success
14. Reward yourself
